# Roscovitine enhances all-*trans* retinoic acid (ATRA)-induced nuclear enrichment of an ensemble of activated signaling molecules and augments ATRA-induced myeloid cell differentiation

**DOI:** 10.18632/oncotarget.27508

**Published:** 2020-03-24

**Authors:** Asif Rashid, Xin Duan, Feng Gao, Mengsu Yang, Andrew Yen

**Affiliations:** ^1^ Department of Biomedical Sciences, City University of Hong Kong, Kowloon, Hong Kong SAR, People’s Republic of China; ^2^ Department of Biomedical Sciences, Cornell University, Ithaca, NY, USA; ^3^ The Sixth Affiliated Hospital, Sun Yat-sen University, Guangzhou, People’s Republic of China

**Keywords:** ATRA, roscovitine, APL, HL-60, Lyn

## Abstract

Although ATRA represents a successful differentiation therapy for APL, it is largely ineffective for non-APL AMLs. Hence combination therapies using an agent targeting ATRA-regulated molecules that drive cell differentiation/arrest are of interest. Using the HL-60 human non-APL AML model where ATRA causes nuclear enrichment of c-Raf that drives differentiation/G0-arrest, we now observe that roscovitine enhanced nuclear enrichment of certain traditionally cytoplasmic signaling molecules and enhanced differentiation and cell cycle arrest. Roscovitine upregulated ATRA-induced nuclear c-Raf phosphorylation at S259 and S289/296/301. Nuclear c-Raf interacted with RB protein and specifically with pS608RB, the hinge region phosphorylation controlling E2F binding and cell cycle progression. ATRA-induced loss of pS608RB with cell cycle arrest was associated with loss of RB-sequestered c-Raf, thereby coupling cell cycle arrest and increased availability of c-Raf to promote differentiation. Part of this mechanism reflects promoting cell cycle arrest via ATRA-induced upregulation of the p27 Kip1 CDKI. Roscovitine also enhanced the ATRA-induced nuclear enrichment of other signaling molecules traditionally perceived as cytoplasmic promoters of proliferation, but now known to promote differentiation; in particular: SFKs, Lyn, Fgr; adaptor proteins, c-Cbl, SLP-76; a guanine exchange factor, Vav1; and a transcription factor, IRF-1. Akin to c-Raf, Lyn bound to RB, specifically to pS608RB. Lyn-pS608RB association was greatly diminished by ATRA and essentially lost in ATRA plus roscovitine treated cells. Interestingly Lyn-KD enhanced such ATRA-induced nuclear signaling and differentiation and made roscovitine more effective. ATRA thus mobilized traditionally cytoplasmic signaling molecules to the nucleus where they drove differentiation which were further enhanced by roscovitine.

## INTRODUCTION

All-*trans* retinoic acid (ATRA), a retinoid metabolite of vitamin A, regulates gene expression [1] in a number of physiological processes, including morphogenesis, vision, growth, metabolism, differentiation and cellular homeostasis [2]. For cancer chemotherapy, ATRA is prominent as a differentiation-inducing therapeutic for acute promyelocytic leukemia (APL) [3, 4], which is a FAB (French American British classification) M3 subtype of acute myeloid leukemia (AML). APL is cytogenetically characterized by a t (15;17) (q22; q12) translocation that results in a PML-RARα fusion protein seminal to the disease [5]. The classical paradigm of ATRA-induced differentiation in leukemia cells focuses on RARα and retinoid X receptors, which are transcription factors activated by binding to their ligands. However, other signaling pathways, particularly mitogen-activated protein kinase (MAPK), have been found to be necessary for RAR and RXR to transcriptionally activate and induce differentiation and G1/G0 cell cycle arrest [6–8]. The Raf/Mek/Erk axis is imbedded in the ATRA-induced signalsome which also includes Src family kinases Fgr and Lyn, PI3K, c-Cbl, SLP-76, Vav1, 14-3-3 and KSR1, plus transcription factors AhR and IRF1 [9–12].

HL-60 cells have been an archetype model for analyzing effects of ATRA *in vitro*. HL-60 cells are lineage bipotent myelo-monocytic precursors established from the peripheral blood of a patient retrospectively diagnosed with acute myeloblastic leukemia (FAB M2) [13]. ATRA induces these cells to undergo myeloid differentiation and G0 cell cycle arrest that depends on a sustained MAPK pathway signal with up-regulation and unanticipated translocation of c-Raf to the nucleus [14, 15]. In the nucleus, c-Raf interacts with the RB tumor suppressor protein [14, 16, 17]. RB is considered a master regulator of the cell cycle that becomes progressively phosphorylated with G1/S/G2 progression. Hypo-phosphorylated RB sequesters the E2F transcription factor that is released with RB phosphorylation to transcriptionally activate genes needed for S-phase entry. Phosphorylation at serine 608 (pS608 RB) is a phosphorylation event signifying a conformational change associated with release of E2F [18]. ATRA-induced RB hypo-phosphorylation requires both RAR/RXR activation to cause G1/G0 arrest [19]. ATRA-induced arrest reflected down-regulation of cyclin E, associated with cyclin dependent kinase inhibitor (CDKI) p27(Kip1) up-regulation [20].

The non-receptor tyrosine kinase Src family of kinases (SFKs) is a group of enzymes that regulate MAPK pathway signaling associated with multiple cellular processes including migration, adhesion, invasion, survival, proliferation and differentiation [21, 22]. Src kinase is the prototypical member of the SFKs, with a total of 8 members expressed in mammalian cells (Src, Blk, Fgr, Fyn, Yes, Hck, Lck and Lyn) [23]. Lyn has been found to be the primary active SFK expressed in AML cells [24, 25]. However, in the ATRA responsive HL-60 non-APL AML cell line, expression of both Lyn and Fgr protein-tyrosine kinases (PTKs) are inducible and tyrosine-phosphorylated [26].

Vav1 is a guanine nucleotide-exchange factor (GEF) that regulates MAPK pathway signaling. Its physiological expression is restricted to hematopoietic systems [27] and up-regulated by ATRA in APL-derived promyelocytes [28]. In malignant promyelocytes, Vav1 interacts with both cytoplasmic and nuclear signaling molecules and participates in interconnected networks regulating the different aspects of ATRA-induced differentiation of APL-derived cells [29]. ATRA drives Vav1 expression and increases association of Vav1 and c-Raf, putatively promoting sustained MAPK pathway activation, cell cycle arrest and differentiation [30]. Vav has been found to be needed for myelopoiesis in knockout mice [31, 32]. In ATRA-induced differentiation of leukemia cells, Vav1 also interacts with PU.1, recruiting it to the promoter to transcriptionally activate expression of the CD11b differentiation marker [33]. IRF is the transcription factor known to be the primary effector of interferon action. It is known to collaborate with ATRA [34]. Like Vav1, IRF-1 also enhanced Raf/Mek/Erk activation and promotes ATRA-induced differentiation and cell cycle arrest [10].

Roscovitine is a purine analogue that inhibits the activity of cyclin-dependent kinases (CDKs) by targeting their ATP-binding pockets [35]. The antitumor activity of roscovitine was demonstrated in various carcinoma cell lines, including nasopharyngeal, ovarian, colon, osteosarcoma, breast, lung and testicular [36, 37]. Roscovitine has been shown to potentiate the effects of other drugs in various hematological diseases. It, for example, synergistically collaborated with high dose farnesyltransferase inhibitor (FTI) to induce caspase-3 activation in HL-60 promyelocytic leukemia cells [38]. While there are reports on roscovitine and apoptosis, there is nothing to our knowledge on regulation of differentiation. While it was originally conceived of as a CDK inhibitor, we now show here that it has other activity and novel downstream targets other than just CDKs. This enhances its interest in chemotherapy and, as we now report, particularly for ATRA-based differentiation induction therapy, which is an entirely novel mechanism for this drug that reveals novel therapeutic vulnerabilities as well as basic molecular mechanistic features of ATRA-induced differentiation of leukemic cells.

In the present study, we found that ATRA caused nuclear enrichment of a number of traditionally cytosolic signaling molecules that earlier reports implicated in the MAPK pathway signaling that drives ATRA-induced differentiation. Roscovitine enhanced the effect of ATRA. Roscovitine enhanced the ATRA-induced nuclear enrichment of c-Raf and the Lyn and Fgr SFKs. RB specifically, pS608 RB, interacted with nuclear c-Raf and Lyn. ATRA plus roscovitine co-treatment diminished the amount of RB bound to c-Raf and Lyn enhancing the availability of freed c-Raf and Lyn in nucleus. We found that ATRA-induced p27Kip1 expression, suppression of cyclin E1 and Cdk2 phosphorylation and loss of pS608 RB were enhanced by roscovitine. Expression of several adaptor proteins, c-Cbl, SLP-76, a guanine exchange factor, Vav1, and the transcription factor, IRF-1 were likewise enriched by ATRA in the nucleus with enhancement by roscovitine. We generated a shRNA Lyn knockdown (shLyn) stable transfectant (Lyn KD), which essentially expressed no detectable pY416 Src, indicating that the phosphorylated nuclear SFK was Lyn and not Fgr. Lyn knockdown augmented certain roscovitine enhancements of ATRA effects on nuclear signaling and cell cycle regulatory molecules. Interestingly Lyn KD did not much modify ATRA effects on c-Raf or its pS289/296/301 form, although it did enhance ATRA-induced p27Kip1 up-regulation as well as down-regulation of pT160 and pY15 Cdk2 and down-regulation of RB and pS608 RB. These effects were associated with enhanced differentiation/cell cycle arrest. Combined ATRA/roscovitine therapy thus enriches an ensemble of canonically cytoplasmic signaling molecules in the nucleus and promotes ATRA-induced differentiation of HL-60 cells. The novel activation of the signaling molecules and translocation to the nucleus during ATRA-induced differentiation is enhanced by roscovitine with concomitant enhancement of induced differentiation. This suggests the potential use of combined therapy with roscovitine in AML patients. Roscovitine is already in clinical trial for patients with advanced solid tumors, but our results suggest that it may be useful in combination with ATRA for differentiation therapy of AML patients.

## RESULTS

### Roscovitine enhances ATRA-induced nuclear enrichment of c-Raf and ATRA regulation of c-Raf/RB and pS608RB complexes

**Figure 1:**
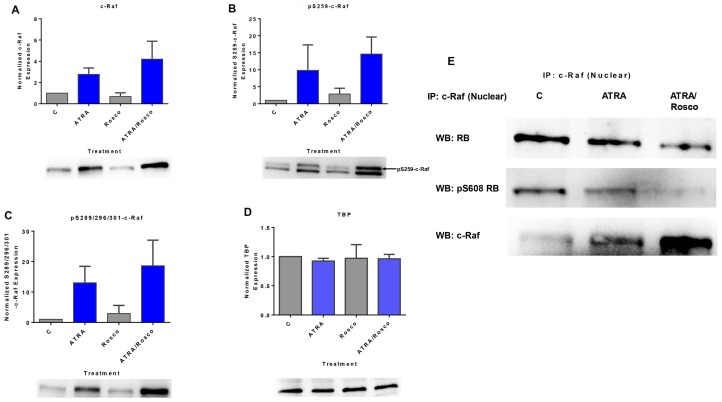
Roscovitine enhances the amount of ATRA-induced phosphorylated c-Raf and phosphorylated c-Raf in the nucleus modulates the RB protein functions. (**A**–**C**) Western blot of c-Raf and its phospho-regulatory residues in HL-60 cells cultured with ATRA for 72 h showed that ATRA upregulated nuclear c-Raf, pS259 and pS289/296/301 c-Raf expression and co-treatment with ATRA plus roscovitine further increased of c-Raf and its active phosphorylation sites, pS259 and pS289/296/301, compared to ATRA alone. (**D**) TATA binding protein (TBP) is the loading control. (**E**) c-Raf immunoprecipitates probed for RB or S608 RB show that roscovitine enhances ATRA-induced downregulation of the amount of nuclear c-Raf complexed with RB and specifically with its serine 608 phosphorylated form (pS608 RB). An equal amount of pre-cleared nuclear lysate was collected 72 h post treatment and incubated overnight with 2.5 μg of the precipitating antibody with magnetic beads and resolved on 12 % polyacrylamide gels. All blots shown are representative of three replicates.

ATRA-induced differentiation is driven by a sustained activation of MAPK signaling that causes the unanticipated nuclear translocation of c-Raf [14, 15]. The nuclear c-Raf phosphorylates transcription factors to enable RAREs to regulate gene transcription needed for ATRA-induced differentiation [8]. We investigated the effect of roscovitine on such signaling seminal to differentiation. Cells were either untreated or treated with ATRA, roscovitine, or ATRA plus roscovitine for 72 h and nuclear lysates were harvested for analysis by Western blotting and immunoprecipitation. ATRA-treated cells showed enhancement of c-Raf expression and its S259 and S289/296/301 phosphorylated forms in the nucleus compared to untreated cells (Figure 1A–1C). Roscovitine enhanced the ATRA-induced accretion of c-Raf and its S259 and S289/296/301 phosphorylated forms in the nucleus. The immunoprecipitation of c-Raf followed by immunoblotting showed that nuclear c-Raf complexed with RB and specifically with pS608 RB-the hinge region phosphorylation that controls E2F binding and cell cycle progression. ATRA reduced the amount of RB and pS608 RB bound with c-Raf, and roscovitine enhanced the reduction in the amount of c-Raf bound with RB and pS608 RB. Roscovitine thus promoted these ATRA-induced effects (Figure 1E). In these cells, ATRA-induced cell cycle arrest was associated with less RB and specifically less pS608 RB [14, 39]. Thus, cell cycle arrest also would result in less c-Raf sequestered with RB, increasing the availability of non-RB-sequestered Raf. This provides a heuristic rationalization for how cell cycle arrest can promote differentiation. Roscovitine is ergo a pharmacological means of evoking the same putative differentiation-promoting effect.

### Roscovitine enhances the ATRA-induced expression of SFKs and pY416 SFKs and pS608 RB hypophosphorylation

SFKs function to promote ATRA-induced differentiation [9, 40]. We therefore tested if roscovitine affected them in a way consistent with driving differentiation. Lyn and Fgr are the predominant SFKs in these cells, and they are up-regulated with ATRA treatment [40]. Given that these ATRA-regulated SFKs participate in inducing differentiation, we characterized the effects of co-treatment with ATRA and roscovitine by measuring their expression and activating phosphorylation. Cells were untreated controls or treated with ATRA, roscovitine or ATRA plus roscovitine. After 72 h of culture, we collected the cell lysate, extracted nuclear protein, and analyzed the expression of Lyn, Fgr, and phospho (Y416)-c-Src proteins.

**Figure 2:**
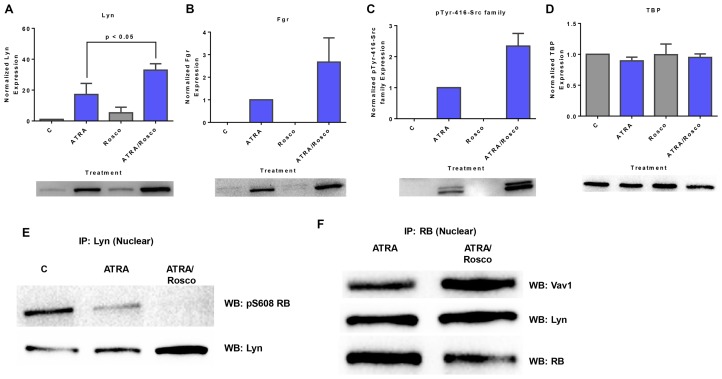
Roscovitine enhances the expression of ATRA-induced enrichment of nuclear Src-family kinase members. Nuclear lysates collected after 72 h of treatment were resolved on 12% polyacrylamide gels. 25 μg protein was loaded per well. (**A–C**) Roscovitine enhances ATRA-induced nuclear Lyn, Fgr and Y416-c-Src expression. *p* < 0.05 comparing ATRA-treated samples to ATRA/roscovitine-treated samples. (**D**) TATA binding protein (TBP) was the loading control. (**E**) Roscovitine augments ATRA-induced reduction of nuclear Lyn interaction with pS608 phosphorylated RB tumor suppressor protein. Co-immunoprecipitation was done using Lyn as bait. (**F**) Nuclear RB binds Lyn in ATRA and ATRA plus roscovitine treated cells. Co-immunoprecipitation was done in treated cells using RB as bait. Vav also binds RB in these cells. An equal amount of pre-cleared nuclear lysate was collected 72 h post treatment and incubated overnight with 1:100 concentration of the precipitating antibody with magnetic beads and resolved on 12 % polyacrylamide gels. All blots shown are representative of three replicates.

Lyn was up-regulated by ATRA, and the expression was further enhanced by co-treatment with roscovitine (Figure 2A). ATRA also induced the expression of Fgr and phospho-Src family (Y416), and co-treatment with ATRA plus roscovitine further enhanced the expression (Figure 2B and 2C). To the best of our knowledge, this study is the first to report the existence of the members of Src-family kinases and phospho-Src family (Y416) in the nucleus of HL-60 human myeloblastic leukemia cells and their nuclear enrichment by treatment with either ATRA alone or ATRA plus roscovitine.

Given that the above results show the presence of SFKs in the nucleus, we explored the association between Lyn and RB. Immunoprecipitation showed that Lyn complexed with RB and in particular its S608 phosphorylated form. ATRA reduced the amount of Lyn complexed with pS608 RB. At the same time Lyn expression in the nucleus was enhanced by ATRA in addition to gains from relieving the amount bound to pS608 RB. Roscovitine enhanced these ATRA-induced effects. Roscovitine thus again potentiated ATRA effects, but it did not cause such effects by itself (Figure 2E). While Lyn binding to pS608 RB was greatly diminished in ATRA treated cells and essentially lost in ATRA plus roscovitine treated cells, Lyn binding to RB was detectable in both (Figure 2F), consistent with preferential binding to non-pS608 phosphorylated RB in the treated cells. Interestingly, like Lyn, Vav likewise binds RB.

### ATRA plus roscovitine co-treatment enhances nuclear VAV1 expression

**Figure 3:**
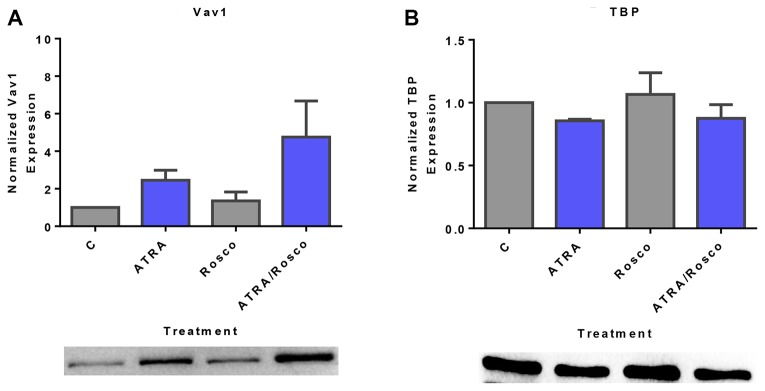
Roscovitine increases ATRA-induced nuclear expression of Vav1. (**A**) Western blots of nuclear lysate shows that ATRA enhances the relative expression of nuclear Vav1 compared to untreated cells and ATRA/roscovitine treated HL-60 cells further increases the level of Vav1 compared to ATRA alone at 72 h. (**B**) TATA binding protein (TBP) was the loading control. All blots shown are representative of three replicates.

Vav1 is a GEF found in both the cytoplasm and nuclear compartments and is the only member of the Vav family expressed in hematopoietic cells [41]. Vav was identified as a component of the cytoplasmic signalsome that drives differentiation [29]. We explored whether Vav was regulated by ATRA and roscovitine as were the related signaling molecules, c-Raf and Lyn, which were also signalsome components. Cells were untreated controls or treated with ATRA, roscovitine or ATRA plus roscovitine. After 72 h of culture, we collected the cell lysate, extracted nuclear protein, and analyzed the expression of Vav. ATRA alone up-regulated nuclear Vav1 expression, and co-treatment with roscovitine caused further nuclear enrichment (Figure 3A). We next searched for Vav partners of regulatory significance in the nucleus. Using immunoprecipitation with RB as bait, we also noted a novel Vav1-RB interaction in the nucleus (Figure 2F). We infer that ATRA causes more Vav in the nucleus where it bound RB. Roscovitine enhanced the ATRA-induced increased Vav in the nucleus, To the best of our knowledge, this result is the first finding that ATRA alone and co-treatment with ATRA plus roscovitine enhanced Vav1 expression in the nucleus where it associated with RB.

### Roscovitine enhances ATRA-induced enrichment of c-Cbl and SLP-76 in the nucleus

**Figure 4:**
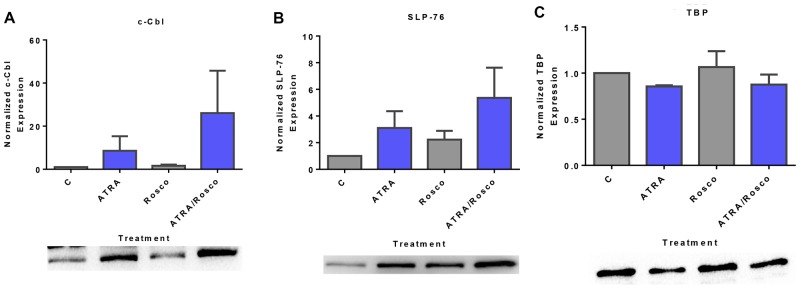
Expression of adaptor proteins (c-Cbl, SLP-76) in the nucleus is enhanced by ATRA and roscovitine. (**A**, **B**) Western blots of nuclear lysate show that ATRA enhances the expression of c-Cbl and SLP-76 compared to untreated cells and co-treatment with ATRA and roscovitine further increased their expression compared to ATRA alone at 72 h. (**C**). (**B**) TATA binding protein (TBP) was the loading control. All blots shown are representative of three replicates.

c-Cbl and SLP-76 are adaptor proteins that also activate sustained MAPK signaling to facilitate ATRA-induced cell differentiation [11, 12, 42]. We explored the effect of ATRA and roscovitine on these signaling adaptor molecules known to be functionally related to c-Raf, Lyn, and Vav. Expression of c-Cbl and SLP-76 are known to drive ATRA-induced differentiation. Cells were untreated controls or treated with ATRA, roscovitine or ATRA plus roscovitine. After 72 h of culture, we collected the cell lysate and extracted nuclear protein for Western blotting. ATRA increased nuclear c-Cbl expression, and roscovitine enhanced the increase (Figure 4A). Similarly, ATRA increased nuclear SLP-76 expression, and adding roscovitine further enhanced expression compared with ATRA alone (Figure 4B). Roscovitine thus had widespread effects on signaling molecules that promote ATRA-induced differentiation.

### Roscovitine augments ATRA-induced nuclear IRF-1 expression

**Figure 5:**
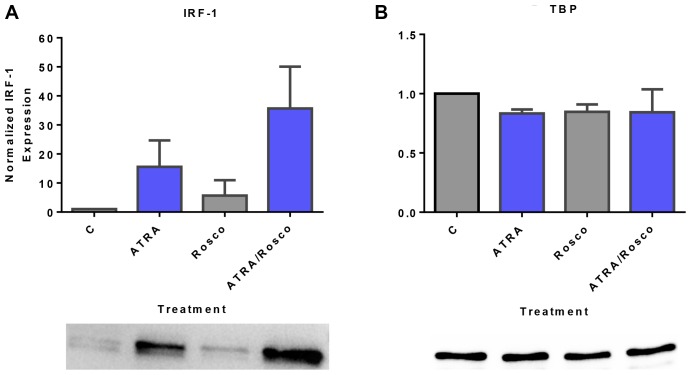
Roscovitine enhances ATRA-induced nuclear enrichment of IRF-1. (**A**) Western blots of nuclear lysate show that ATRA enhances the relative expression of IRF-1 compared to untreated cells and cells co-treated with ATRA plus roscovitine further increases the level of IRF-1 compared to ATRA alone at 72 h. (**B**) TATA binding protein (TBP) was the loading control. All blots shown are representative of three replicates.

IRF-1 is a transcription factor found to have a signaling function in the signalsome that promotes ATRA-induced differentiation [34]. Given that ectopically expressed IRF-1 enhanced Raf/Mek/Erk activation and ATRA-induced cell differentiation and G1/G0 arrest [10], we explored if like other signaling molecules in the signalsome it was subject to regulation by ATRA and roscovitine. Cells were untreated or treated with ATRA and ATRA plus roscovitine for 72 h and harvested to generate nuclear lysates to analyze for nuclear IRF-1 expression by Western blotting. ATRA increased the amount of IRF-1 in the nucleus compared to untreated cells. Adding roscovitine further enhanced IRF-1 expression compared to ATRA alone (Figure 5A). Roscovitine effects thus extended to the IRF-1 transcription factor in addition to classical signal transduction molecules.

### Roscovitine enhances ATRA-induced changes in certain canonical cell cycle regulators: p27/cyclin E1/Cdk2/RB pathway

**Figure 6:**
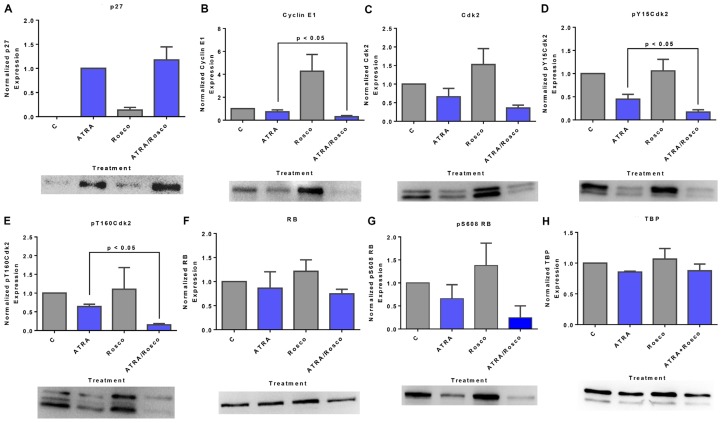
Roscovitine effects on ATRA-induced changes in nuclear expression of G1/G0 regulatory molecules: p27/cyclin E1/Cdk2/RB pathway. (**A**) Western blot of p27Kip1 in cells cultured for 72 h showed that ATRA enhanced nuclear p27Kip1 level, and cells co-treated with ATRA and roscovitine modestly further upregulated the p27Kip1 expression. (**B**) Roscovitine further decreased ATRA-induced reduction of nuclear cyclin E1 expression in these cells. (**C–E**) Roscovitine diminishes ATRA-induced downregulation of nuclear CDK2 and specifically its pY15CDK2 and pT160CDK2 phosphorylated forms. (**F**, **G**) ATRA plus roscovitine downregulates total RB and pS608 phosphorylated RB. Roscovitine enhanced ATRA-induced reduction of RB phosphorylated at pS608 site. Surprisingly, at the same dose, roscovitine alone enhances nuclear cyclin E1 and CDK2 expression. *p* < 0.05 comparing ATRA-treated samples to ATRA/Roscovitine-treated samples. (**H**) TATA binding protein (TBP) was the loading control; a minor artifact caused during image capture can be seen. All blots shown are representative of three replicates.

Given the effects of roscovitine on signaling and the unanticipated nuclear translocation and association with RB of these molecules, we explored the effects of roscovitine on classical cell cycle regulatory molecules. Cells were untreated or treated with ATRA, roscovitine or ATRA plus roscovitine and harvested after 72 h for Western blot analysis of nuclear lysates probed for the cell cycle regulators, p27Kip1, Cyclin E1, Cdk2, pY15 and pY160 phosphorylated CDK2, RB, and pS608 RB. The p27Kip1 cyclin dependent kinase inhibitor (CDKI) plays a key role in determining the onset of the S-phase [43]. ATRA induced p27Kip1 expression consistent with the G1/G0 cell cycle arrest known to occur [44] and adding roscovitine slightly enhanced this (Figure 6A). p27Kip1 is known to target the cyclin E1/Cdk2 complex. We analyzed the expression of total Cdk2, phospho-Y15Cdk2, phospho-T160Cdk2, and cyclin E1 and found that co-treated cells down-regulated cyclin E1 and Cdk2, notably both its Y15Cdk2 and T160Cdk2 phosphorylated forms. Roscovitine generally enhanced the effects of ATRA on these cell cycle regulators (Figure 6B–6E). p27Kip1, cyclin E, and cdk2 are canonical regulators of RB, where activated cyclin E1-Cdk2 phosphorylates RB to cause S-phase entry [43]. We analyzed the expression of total RB and pS608-RB status in these cells. Consistent with other reports [14, 16, 39, 45–47], ATRA promoted the down-regulation of total RB and diminished the amount of pS608-RB, and co-treatment with roscovitine enhanced this (Figure 6F and 6G). Thus, roscovitine enhanced ATRA-induced effects on G1/G0 cell cycle arrest through regulatory proteins of the p27/cyclin E1/Cdk2/RB pathway. Notably, roscovitine itself had effects on such cell cycle regulators. It induced the expression of cyclin E1, Cdk2, pY15Cdk2, and pT160Cdk2. Based on the above results, roscovitine affects ATRA-regulated signaling molecules that drive induced differentiation and also ATRA-regulated cell cycle regulatory molecules that control G1/G0 arrest.

### Lyn knockdown enhances ATRA and roscovitine-induced nuclear protein expression changes

**Figure 7:**
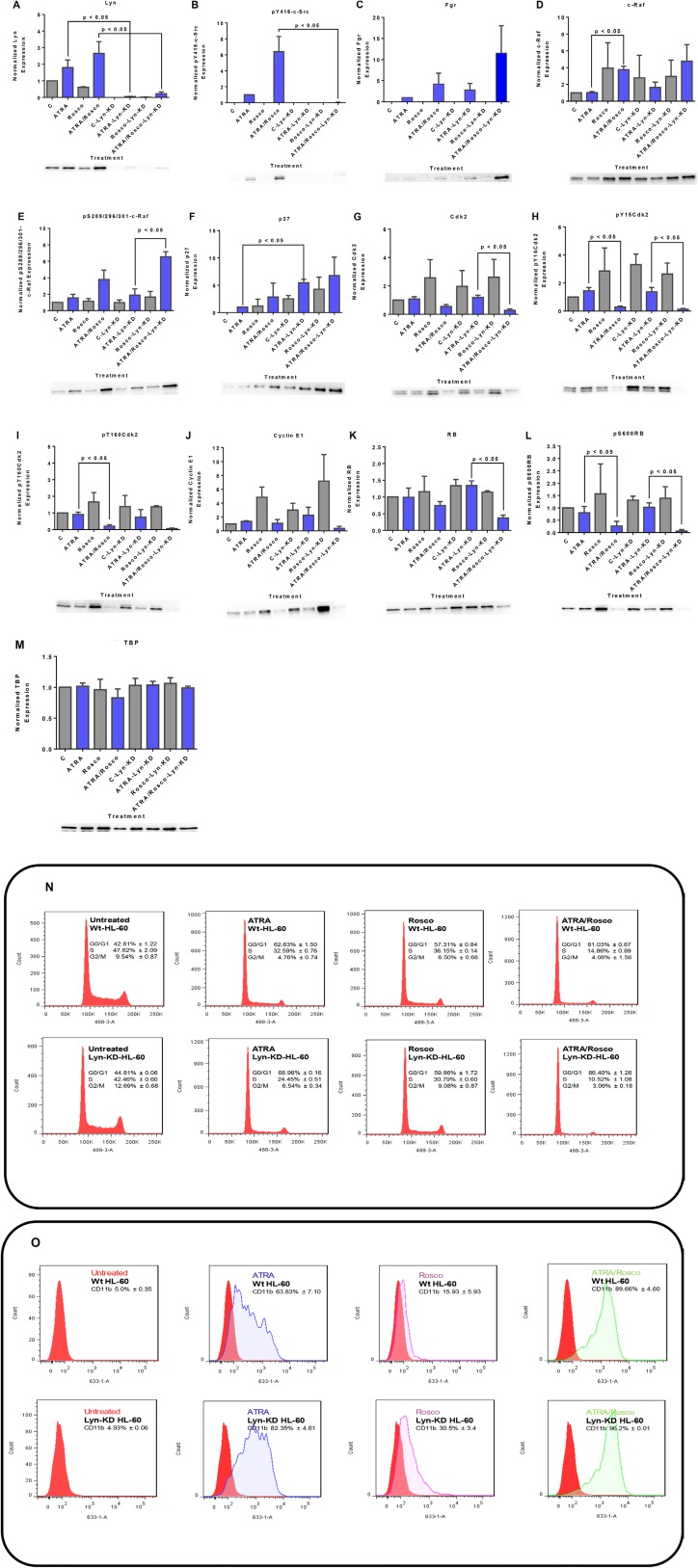
Western blots of nuclear lysate, CD11b and DNA histograms show that Lyn knockdown enhances ATRA-roscovitine induced gene expression and myeloid differentiation. (**A**, **B**) ATRA and ATRA plus roscovitine co-treated Lyn KD cells caused a modest increase in Lyn and the phospho-residue, pY416-c-Src, expression. (**C**) Lyn knockdown enhances Fgr expression in Lyn KD cells co-treated with ATRA and roscovitine. (**D**, **E**) Lyn knockdown enhances c-Raf and phospho- S289/296/301-c-Raf expression in Lyn KD cells co-treated with ATRA and Roscovitine (**F**). p27Kip1 protein expression upregulates in Lyn KD cells co-treated with ATRA and roscovitine (**G**–**J**). Total Cdk2, phospho-Cdk2Y15, phospho-Cdk2T160 and cyclin E1 downregulates in Lyn KD cells co-treated with ATRA and roscovitine (**K**, **L**). Cotreatment of Lyn KD cells also deceases total RB expression and induced hypophosphorylation at serine 608 (S608) of RB (**M**). TATA binding protein (TBP) was used as loading control. p < 0.05 comparing ATRA-treated samples to ATRA/Roscovitine-treated samples of either wild-type and Lyn-KD cells. (**N**) Cell cycle distribution showing the percentage of cells in G1/G0, S and G2/M was analyzed using flow cytometry with propidium iodide. ATRA/roscovitine-induced differentiation of Lyn KD stable cells marked by G1/G0 cell cycle arrest was enhanced compared to parental wild type cells. (**O**) CD11b expression assessed by flow cytometry with an APC-conjugated antibody. Lyn KD cells show enhanced CD11b expression in response to ATRA when compared to wild-type cells. Also, a difference in CD11b expression was found between ATRA/roscovitine-treated parental wild type and Lyn KD cells. All experiments shown are representative of three replicates..

The data above implicate Lyn with a prominent role in mediating the effects of ATRA and roscovitine, but there are conflicting reports on how Lyn is involved. Miranda *et al.* [48] reported that the inhibition of Src family kinases enhances ATRA-induced myeloid cell differentiation. In contrast we reported that putative SFK inhibitors could in fact enhance the signaling that drives differentiation and enhance differentiation [9, 40]. It is ergo not clear how SFK activity regulated ATRA-induced differentiation, although the significant engagement of Lyn in the action of ATRA and roscovitine was indicated by our earlier data. We explored Lyn function in mediating the effects of ATRA and roscovitine by shRNA knockdown targeting Lyn, in this case obviating potential off target effects of pharmacological agents. We constructed a pLKO.1-LynshRNA expression vector and generated stable transfectants expressing shRNA targeting Lyn (shLyn). The knockdown efficiency was assessed by Western blot. Stable transfectants expressing the shRNA targeting Lyn essentially lost all Lyn expression. Nor could ATRA or ATRA plus roscovitine induce Lyn expression. After 72 h, ATRA and ATRA plus roscovitine treatment of shLyn stable cells no longer up-regulated expression of Lyn. No phospho residue pY416-c-Src was detectable in the nucleus of the stable transfectants (Figure 7A and 7B). Interestingly, Lyn knockdown enhanced up-regulation of nuclear Fgr by both ATRA and ATRA plus roscovitine. (Figure 7C)**.** Since Lyn and Fgr are essentially the only two SFKs in these cells, and Lyn KD resulted in loss of pY416 SFK, then the pY416 SFK phosphorylation must reflect only Lyn phosphorylation at Y416 SFK activation site. Fgr ergo is not Y416 phosphorylated in response to ATRA or to roscovitine. Interestingly loss of one SFK has enhanced the induced expression of the other, suggesting compensatory cross talk between Lyn and Fgr.

c-Raf is a kinase known to drive the differentiation process and to collaborate with Fgr [9]. As in the case of Fgr, Lyn KD enhanced ATRA/roscovitine-induced up-regulation. Likewise, up-regulation of pS289/296/301-c-Raf was enhanced by Lyn KD. Interestingly, as for Fgr, the Lyn KD itself caused increased c-Raf in the nucleus (Figure 7D and 7E).

p27Kip1 is a CDKI cell cycle regulator, and we sought evidence that it regulated ATRA and roscovitine effects on canonical cell cycle regulatory molecules. ATRA induced up-regulation of p27Kip1 expression where addition of roscovitine enhanced this; and Lyn KD resulted in further up-regulation. The Lyn KD thus enhanced the induced up-regulation of p27Kip1 (Figure 7F). In contrast Lyn KD did not significantly further affect expression of cyclin E nor Cdk2 or its pY15 and pT160 forms in ATRA/roscovitine treated cells. (Figure 7G–7J). RB is downstream of the p27Kip1 CDKI, E cyclin, and CDK2 axis; and Lyn KD enhanced ATRA/roscovitine-induced down regulation of RB, but without much further effect on the ATRA/roscovitine-induced loss of pS608 RB (Figure 7K and 7L).

The above reported data on the effects of Lyn knock down as well as roscovitine on the differentiation promoting signaling and cell cycle regulators motivated us to seek evidence that they promote cell cycle arrest and differentiation. Cell cycle arrest at G1/G0 is a feature of differentiation. Cell cycle phase distribution was measured by flow cytometry in untreated, ATRA, roscovitine and ATRA plus roscovitine treated wild-type and Lyn KD cell populations. We found that ATRA treated Lyn KD cells showed progressively more G1/G0 enrichment than wild type cells, consistent with retardation of growth, and adding roscovitine to ATRA further enhanced the accumulation of these cells in G1/G0 compared to ATRA alone (Figure 7N). CD11b is an integrin receptor subunit that is a differentiation marker. We also found that ATRA treated Lyn KD stable transfectants showed more CD11b expression than wild type and adding roscovitine to ATRA modestly enhanced CD11b expression (Figure 7O). So roscovitine enhancement of ATRA ergo was not lost with loss of Lyn. The changes observed in nuclear signaling molecule responses to ATRA and roscovitine due to Lyn KD are thus associated with enhancement of cell cycle arrest and differentiation.

### Hierarchical clustering based on nuclear protein expression and activation in HL-60 Wt and HL-60 Lyn-KD cells

Hierarchical clustering analysis for an ensemble of known cell cycle regulatory molecules and canonical growth factor receptor regulated cytosolic signaling molecules now found in the nucleus was performed to identify coupling relationships that betray regulatory pathways driving cell differentiation induced by ATRA and enhanced by addition of roscovitine.

In the wt parental cells, ATRA and roscovitine treatments reveal two main clusters determined by absence or presence of ATRA. Each of these resolves into cells without roscovitine or with roscovitine. The main determinant of variance is hence ATRA which is modified by roscovitine, so biologically roscovitine is just a modifier of a cellular response to ATRA, which is the main driver.

**Figure 8:**
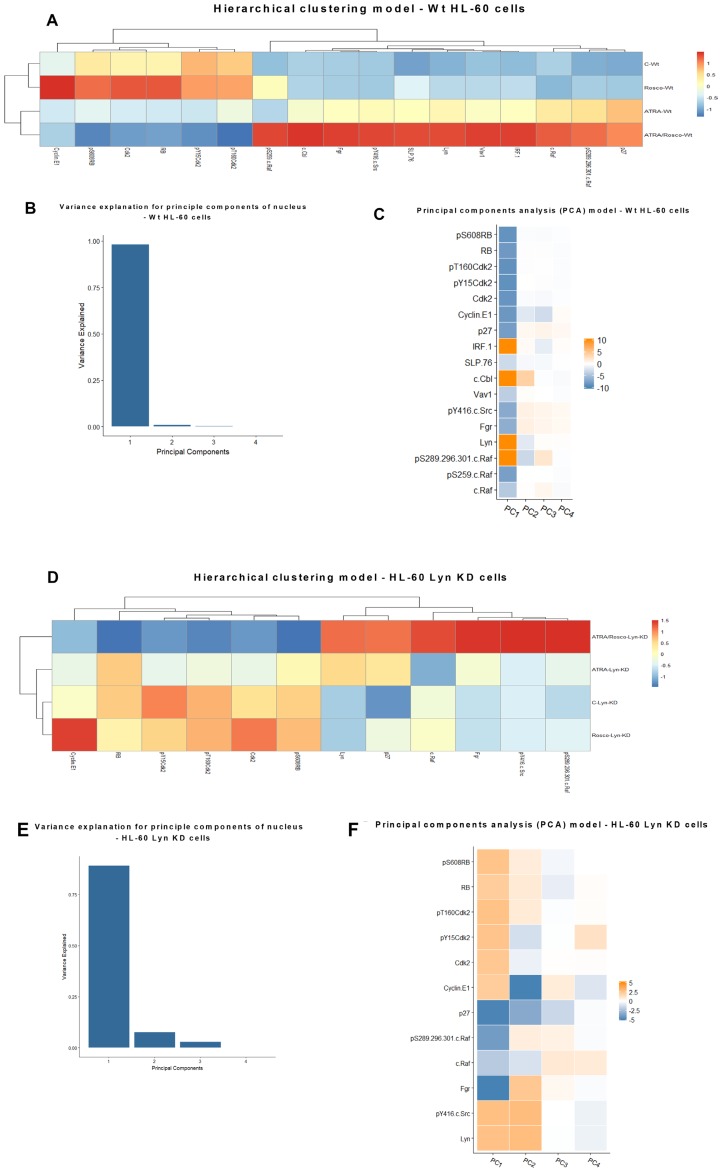
Hierarchical clustering and principal components analysis (PCA) of nuclear protein expression/activation in HL-60 wt and Lyn KD cells. (**A**) Clustering based on the expression and activation of nuclear molecules for HL-60 cells either untreated or treated with ATRA, roscovitine or ATRA plus roscovitine, was performed using the ‘pheatmap’ function available in the R package. (**B**) Contribution by principal components for HL-60 wt cells. (**C**) Molecular contributors of principal components and their coupling (coupling shown by color similarity) for HL-60 wt cells. (**D**) Hierarchical clustering of nuclear protein expression/activation for HL-60 Lyn KD cells. Clustering based on the expression and activation of nuclear molecules for HL-60 Lyn KD cells either untreated or treated with ATRA, roscovitine or ATRA plus roscovitine, was performed using the ‘pheatmap’ function available in the R package. (**E**) Contribution by principal components for HL-60 Lyn KD cells. (**F**) Molecular contributors of principal components and their coupling (by color) for HL-60 Lyn KD.

The ensemble of measured regulatory molecules responding to treatment segregates into two main clusters, cell cycle regulators and cell signaling differentiation regulators. Confirming the fidelity of the analysis to known cell cycle biology, the cell cycle regulators show the anticipated relationships, except for one revealing detail. In this cell cycle cluster, pS608 RB is coupled to CDK2, which is known to phosphorylate RB, and Cyclin E1 is coupled to both of these, which reflects the classical Cyclin E1 regulation of CDK2 [43]. Somewhat surprisingly the putative canonical inhibitory and enhancing phosphorylation events, pY15 and pT160, of CDK2 are coupled and co-regulated (Figure 8A). Significantly the CDKI, p27 Kip1, is not in this cluster, although it is a classical inhibitor of CDK2 and mediates cell cycle arrest as is occurring under the influence of ATRA and ATRA plus roscovitine. The signaling regulators segregate into three discernible clusters that are followers that co-vary together with pS259-c-Raf as their driver. Notably pS259-c-Raf Raf is the driver for these three signaling subclusters, consistent with the postulated regulatory significance of pS259 Raf and its nuclear translocation in ATRA-induced differentiation [9]. Of the three signaling molecule clusters, one contains Fgr and pY416 Src, which in this process we biochemically established as linked [9], another contains the Lyn SFK, and the third includes pS289/296/301-c-Raf coupled to p27 Kip1 (Figure 8A). So, the p27 Kip1 surprisingly appears in the group of signaling molecules covarying with c-Raf/phospho-c-Raf. Interestingly each of these signaling subclusters also has an entity that goes from essentially not expressed in untreated cells to clearly expressed in ATRA treated cells, namely Fgr, IRF-1, and p27 Kip1. And addition of roscovitine enhances these up-regulations. The coupling of the p27 Kip1 CDKI with a putative major signaling regulator of differentiation suggests how signaling driving differentiation is coupled to driving cell cycle arrest. Indeed, the p27 Kip1 gene promoter is Sp1 regulated [49] where the Sp1 transcription factor is a classical responder to Raf/Mek/Erk axis MAPK pathway signaling [50]. p27 kip1 may thus be a molecular link connecting differentiation signaling to cell cycle arrest. Principal components analysis (PCA) revealed essentially only one principal component (Figure 8B) where pS289/296/301 c-Raf, Lyn, c-Cbl, and IRF1 were coupled as one major contributor and p27 Kip1, Cyclin E1, Cdk2, pY15 Cdk2, pT160 Cdk2, RB and pS608 RB were coupled as the other major contributor. The cell cycle regulators including p27 Kip1 appear coupled as a group (Figure 8C).

The Lyn SFK is a putatively important regulator of signaling activation that drives ATRA-induced differentiation. Historically it is a known regulator of many of the signaling molecules [9, 42] implicated here in driving ATRA-induced differentiation. A Lyn shRNA KD was created to experimentally probe the effect of this disruption on the ATRA-induced coupling of regulatory molecules to gain molecular mechanistic insights into the mechanism of induced cell differentiation. The Lyn KD disrupts certain features revealed by the hierarchical clustering analysis, but the gross features are largely conserved albeit with some potentially notable changes. Segregation by treatment is largely as it was with the effect of ATRA modified by roscovitine. Segregation by molecular entities again resolves into two main clusters, namely cell cycle regulators and signaling molecules driving differentiation. The clustering of coupled cell cycle regulators is unaffected by the Lyn KD. Although the signaling molecules still cluster, the hierarchical structure within this cluster is affected. Interestingly we note that pS289/296/301-c-Raf, which was with c-Raf and p27 kip1 in a cluster distinct from the cluster containing Fgr and pY415 SFK in parental cells, has divorced to cluster with Fgr and pY416 SFK in Lyn KD cells (Figure 8D). The coupling between Fgr and pY416 SFK is conserved comparing parental and Lyn KD cells. p27 kip1 coupling to signaling molecules is ergo altered, too. Principal components analysis revealed several principal components (Figure 8E) with one dominant one where p27 Kip1 and Fgr were coupled as one major contributor and pT160 CDK2 and pS608 RB were coupled as the other major contributor. P27 Kip1 was ergo coupled differently compared to wt parental cells (Figure 8F). These changes may contribute to the greater efficacy of ATRA and ATRA plus roscovitine to induce differentiation of Lyn KD cells. The collective changes reflect a global signaling enhancement that apparently drove enhanced differentiation.

## DISCUSSION

Retinoic acid differentiation therapy has been successfully used to treat acute promyelocytic leukemia (APL), which is classified as the M3 subtype of AML in the FAB classification system and accounts for approximately 5%–8% of patients with AML [51], but it has not been effective for the majority of AML. In APL, the cause of disease is thought to arise from a t (15;17) translocation resulting in a PML-RARα fusion protein. ATRA can induce the proteolytic degradation of this fusion protein resulting in the repression of cell proliferation and induction of myeloid cell differentiation [52]. However, some patients relapse, and disease recurrence is associated with resistance to ATRA [53]. This and the fact that ATRA is ineffective at inducing remissions in the majority of AML have stimulated interest in combination therapies using ATRA with other agents. Such therapies hold the promise of both overcoming resistance and mitigating the incidence of retinoic acid syndrome, a potentially fatal cardio-pulmonary pathological sequela of ATRA therapy, by reducing the effective dose of ATRA needed. Much effort has been devoted to identifying novel drugs with specific targets that would increase the therapeutic efficiency of ATRA [54–58]. In a series of studies, we have established that ATRA-induced differentiation and cell cycle arrest of a FAB M2 cell line model requires formation and activation of a macromolecular signaling complex, a signalsome, that incorporates a number of signaling molecules associated with MAPK pathway signal transduction as well as unexpected components, in particular the IRF-1 and AhR transcription factors [10, 57]. These molecular mechanistic insights motivated tests of agents targeting signalsome components. The signalsome results in nuclear events that enable RAR/RXR, ATRA activated transcription factors, to transcriptionally activate their target genes to drive differentiation and cell cycle arrest. For example, our laboratory reported that the SFK inhibitors PP2, dasatinib, and bosutinib modulate MAPK signaling and enhance the therapeutic effects of ATRA in various myeloid leukemia cells [9, 40]. We have also found that AhR ligands, specifically FICZ and VAF347, used with ATRA enhance induced differentiation [57, 58]. There is hence encouragement to find agents that enhance ATRA via gaining insight into novel molecular mechanistic underpinnings of ATRA action. Ultimately such cocktails could render ATRA resistant AML susceptible to differentiation therapy.

In the present work, we reported that roscovitine collaborates with ATRA to cause nuclear enrichment of proteins known to drive differentiation and cell cycle arrest of the t(15;17)-negative HL-60 human myeloblastic leukemia model. An ensemble of traditionally regarded cytosolic signaling molecules was unexpectedly found in the nucleus where their expression or phosphorylation state was regulated by ATRA. Roscovitine was found to target these and enhance effects of ATRA. One of these molecules was c-Raf. ATRA is known to cause its enrichment in the nucleus where it functions in a non-canonical signaling role to target transcription factors that drive differentiation [8]. In the current study, we found that roscovitine enhanced the ATRA effect. HL-60 cells co-treated with ATRA and roscovitine showed increased nuclear c-Raf levels. c-Raf function in various contexts is controlled by site-specific phosphorylation that controls its binding to various other proteins [59]. How roscovitine caused activation of kinases other than CDKs is unknown. In the current study, we see that roscovitine enhanced ATRA-induced nuclear c-Raf phosphorylation at S259 and S289/296/301, which are known to be associated with differentiation [9]. Although nuclear c-Raf phosphorylated at S621 is implicated in myeloid cell differentiation [8], the potential roles of other sites (S259 and S289/296/301) remain to be determined. In the nucleus, the RB tumor suppressor protein is known to be central in cell cycle regulation and by inference differentiation. During the cell cycle progression of untreated HL-60 cells, RB is in the hyper-phosphorylated state but begins to be hypo-phosphorylated in late G2 in ATRA-treated cells [39]. Hypo-phosphorylated RB is only detectable in cells undergoing differentiation [19]. We found that the c-Raf in the nucleus interacted with RB and specifically with pS608 RB. pS608 is the hinge region phosphorylation that controls E2F binding and cell cycle progression. The ATRA-induced loss of pS608 RB with cell cycle arrest is associated with less RB and specifically less pS608 RB bound to Raf, even as the amount of nuclear c-Raf increases. Roscovitine promoted the loss of c-Raf bound with RB. Hence cell cycle arrest with loss of pS608 RB liberated c-Raf from RB and resulted in more c-Raf availability to stoichiometrically favor targets such as transcription factors that drive differentiation. This provides a heuristic mechanistic rationalization coupling cell cycle arrest and differentiation through the availability of non-RB-sequestered Raf.

The members of the Src kinase family such as Blk, Hck, Fgr, Lck, and Lyn are primarily found in hematopoietic cells [60]. Among these, Lyn and Fgr are progressively activated by tyrosine phosphorylation after ATRA treatment of HL-60 cells [26]. In the current study, we found that roscovitine enhanced ATRA-induced enrichment of the SFK members Lyn and Fgr and promoted Y416 phosphorylation in the nucleus. Phosphorylation of Y416 marks activation of SFKs. We observed that whereas Fgr and Lyn are the primary SFKs known in these cells, knocking down Lyn eliminated detectable pY416 SFK indicating that Lyn and not Fgr was the primary phosphorylated SFK in the nucleus of ATRA-treated cells. Given that the above results indicate the presence of SFKs in the nucleus, we searched for Lyn nuclear partners of potential regulatory significance. Through co-immunoprecipitation, we found that Lyn complexed with RB after ATRA or combined ATRA and roscovitine treatment.

The role of Lyn in ATRA-induced differentiation is somewhat enigmatic. We reported that ATRA causes up-regulation of Lyn. However, previous reports show that some SFK inhibitors enhanced the ATRA-induced expression of SFK members, Lyn and Fgr, as well as activated signaling which was associated with promoting cell cycle arrest and differentiation [9, 24, 40]. And Lyn knock down also did this, too. But roscovitine enhances the ATRA-induced increase of Lyn and Fgr in the nucleus and promotes ATRA-induced differentiation. This is paradoxical, however, as it is known that in a variety of cases SFK inhibitors can also act as activators, too [61], suggesting that context dependence may be a partial explanation. Certainly, off target effects of the drugs could have resulted in compensatory effects, too.

We then explored Vav1 expression and found that roscovitine increases ATRA-induced nuclear enrichment of Vav1. Vav1, a guanine nucleotide exchange factor, plays a central role in the activation of MAPK signaling cascade [62] and is associated with downstream expression of the differentiation markers CD38 and CD11b. Brugnoli *et* *al*. [33] reported that the ATRA-induced expression of Vav1 recruits PU.1 to its consensus sequence on the CD11b promoter and ultimately regulates CD11b expression during the late stages of the neutrophil differentiation of APL-derived promyelocytes. To our knowledge, no previous evidence shows that roscovitine regulates Vav1 activity in myeloid cells, but synthesizing these results with ours suggests roscovitine could be promoting this to drive the phenotypic shift characterizing differentiation.

The adaptor proteins c-Cbl and SLP-76 also promote ATRA-induced differentiation and the G1/G0 arrest of HL-60 cells [11, 12, 42]. Shen and Yen [63] showed that c-Cbl interacts with CD38 to enhance ATRA-induced differentiation. This is the first report showing that roscovitine enhances the ATRA-induced nuclear enrichment of c-Cbl and SLP-76. Because these adaptor molecules also support signaling by other chemokines that regulate myeloid differentiation, they may also promote signaling pathways collaborating with ATRA during induced differentiation. A well-established collaborating pathway is interferon enhancement of ATRA-driven differentiation [34]. Indeed, we have observed that ATRA induces IRF-1 expression and ectopic expression of IRF-1 propels ATRA-induced differentiation and arrest of these cells [10]. IRF-1 is the well-known transcription factor implicated in being the primary driver of IFN-γ effects [64]. As with adaptor proteins, roscovitine also drives nuclear IRF-1 expression, augmenting ATRA-induced increases that we previously showed enhance Raf/Mek/Erk activation and promote differentiation and cell cycle arrest [10].

We found that roscovitine enhanced ATRA-induced reduction of cyclin E1, CDK2, pY15 CDK2 and pT160 CDK2, and pS608 RB. Roscovitine enhanced the ATRA-induced increase in p27Kip1 level. The observed ATRA and roscovitine driven reduction in cyclin E1 levels possibly contributes to the following increase in p27Kip1 stability after ATRA-roscovitine treatment because cyclin E-CDK2 complexes can target p27Kip1 for degradation through phosphorylation of Thr187 [65, 66]. RB is the target of CDKs. We found that ATRA induced loss of pS608 RB, which was enhanced by roscovitine. This may have dual effects of enhancing sequestering E2F to cause G1/0 arrest and freeing molecules sequestered by pS608 RB to drive differentiation. RB may thus sequester factors driving differentiation during cell proliferation when S608 is phosphorylated, and sequester factors driving proliferation when S608 phosphorylation is lost and freeing factors that drive differentiation. Hence depending on its phosphorylation state RB may be promoting either proliferation or differentiating by differential sequestration of drivers of these processes.

We also explored Lyn function in mediating the effects of ATRA and roscovitine by shRNA knockdown targeting Lyn and found that Lyn knockdown enhanced up-regulation of nuclear Fgr by both ATRA and ATRA plus roscovitine. Since Lyn KD resulted in loss of pY416 SFK, the pY416 SFK phosphorylation must reflect only Lyn phosphorylation at Y416 SFK activation site. Fgr ergo is not Y416 phosphorylated in response to ATRA or to roscovitine. Interestingly loss of one SFK, Lyn, has enhanced the induced expression of the other, Fgr, suggesting compensatory cross talk between Lyn and Fgr. Our data clearly implicate the Lyn KD effects on nuclear signaling pathways considered seminal to ATRA-induced differentiation and cell cycle arrest.

**Figure 9:**
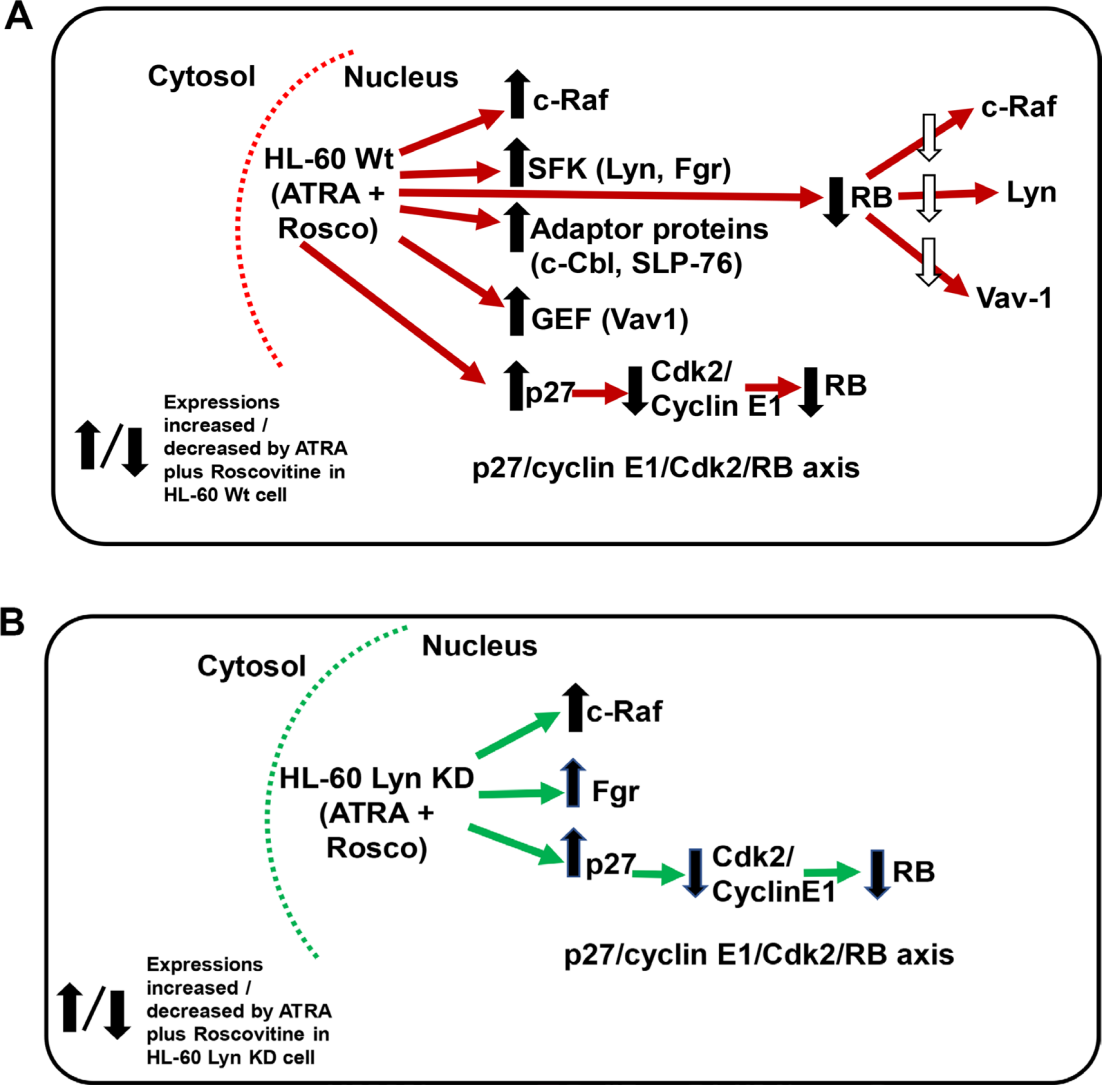
(**A** and **B**) Schematic diagram of ATRA-roscovitine induced modulation of nuclear molecules in wild-type and Lyn-KD HL-60 cells. Red/Green arrow shows flow of either Wt or Lyn KD HL-60 cells. Up/down arrows show effects shown to be affected by adding ATRA plus Roscovitine.

In conclusion, the current study showed that roscovitine exhibits effects beyond its original presentation as a CDK inhibitor (Figure 9A and 9B). Roscovitine may modulate nuclear molecules and enhance the therapeutic effects of ATRA in HL-60 cells. To the best of our knowledge, this study is the first to report that roscovitine potentiates ATRA in inducing myeloid leukemia cell differentiation, the mechanistic insights of which suggests new therapeutic targets to improve the clinical efficiency of ATRA to treat myeloid leukemia.

## MATERIALS AND METHODS

### Cell culture and treatments

HL-60 human myeloblastic leukemia cell lines derived from the original isolates were a generous gift of Dr. Robert Gallagher. They were certified and tested for mycotoxin by Bio-Synthesis (Lewisville, TX, USA) in August 2017. Cells were cultured in RPMI 1640 medium (Invitrogen, Carlsbad, CA) supplemented with 5% heat inactivated fetal bovine serum (Invitrogen, Carlsbad, CA) and 1% antibiotic-antimycotic (Thermo Fisher Scientific, Waltham, MA) in a 5% CO_2_ humidified atmosphere at 37° C. Cell growth and viability was measured with hemocytometer and 0.2% trypan blue (Invitrogen, Carlsbad, CA) exclusion assay.

Four treatment regimens were studied: (1) untreated, (2) All-*trans* retinoic acid (ATRA), (3) Roscovitine (Rosco) and (4) ATRA plus Roscovitine (ATRA/Rosco). ATRA (Sigma, St. Louis, MO) was added from a 5 mM stock solution in 100% ethanol to a final concentration of 1 μM in culture as previously described [19]. Roscovitine from EMD Millipore Corp., (Billerica, MA) was also solubilized in 100% ethanol at 1 mM. A dose response curve assaying cell number and viability over a 72 h course using 1, 2, 4, 6, 8 and 10 μM roscovitine showed that 6 μM dose was at the threshold of overt growth arrest and toxicity. Cells were treated with a final concentration of 6 μM.

### Antibodies and reagents

Antibody for flow cytometric analysis of CD11b (clone ICRF44) conjugated with allophycocyanin (APC) was from BD Biosciences (San Jose, CA). Protein G magnetic beads used for immunoprecipitation were from Millipore (Billerica, MA). Antibodies for western blot probing against TBP, Phospho-c-Raf (S259), Phospho-c-Raf (S289/296/301), SLP-76, Lyn, Fgr, Vav1, RB, HRP anti-mouse and anti-rabbit were from Cell Signaling (Danvers, MA). Cdk2, Phospho-Cdk2 (T160), Phospho-Cdk2 (Y15), Phospho-RB (S608) and Cyclin E1 antibodies were from AbCam (Cambridge, MA). c-Raf and IRF-1 antibodies were from BD Biosciences (San Jose, CA). c-Cbl antibody was from Santa Cruz Biotechnology (CA, USA). NE-PER Nuclear and cytoplasmic extraction reagents were from Pierce Biotechnology (Thermo Scientific, Rockford, IL). Bovine serum albumin (BSA), Triton X-100, protease and phosphatase inhibitors were purchased from Sigma (St. Louis, MO).

### Flow cytometric phenotypic analysis

Immunostaining for CD11b was performed as previously described [63] and fluorescence was detected using a Becton Dickinson LSR II flow cytometer (San Jose, CA). Gating was set to exclude 95% of the untreated wild-type and Lyn KD HL-60 samples. Cell cycle analysis was performed as previously described [63].

### Western blotting and immunoprecipitation

Cells were washed, pelleted and nuclear protein was extracted. The nuclear – cytoplasmic fractionation was done using the NE-PER nuclear and cytoplasmic extraction kit (ThermoFisher Scientific, Rockford, IL) per the manufacturer’s instructions with the addition of protease and phosphatase inhibitors. The purity of the nuclear and cytoplasmic fractionations was assessed using clathrin as a cytoplasmic marker and TATA binding protein (TBP) as a nuclear marker. The nuclear fractions used were verified as TBP positive and clathrin negative by Western blotting.

Protein concentration was determined using the Pierce BCA protein assay (Thermo Scientific, Rockford, IL) according to the manufacturer’s protocol. For immunoprecipitation experiments, equal amounts of protein were pre-cleared with PureProteome protein G magnetic beads (Millipore, Billerica, MA) and then incubated overnight with beads and appropriate antibodies. Bead/antibody/protein slurries were then washed and subjected to standard SDS-PAGE analysis. For western blotting, 25 μg of protein was resolved by SDS-PAGE using 12% polyacrylamide gel. Electro-transfer was done onto PVDF membranes (Millipore, Billerica, MA) at 400 mA. The membranes were blocked in dry nonfat milk before being incubated with the indicated primary antibody overnight at 4° C. Images were captured on a Bio-Rad ChemiDoc XRS Molecular Imager and analyzed using Image J software. Densitometric values for each Western blot band were determined using Image J. The values were then normalized to the loading control for that lane. For scaling in the bar graphs, the lowest normalized value is arbitrarily set to one and the values for other bands normalized to that and shown relative to the lowest value, which was typically the untreated control unless there was no detectable signal then the lowest detectable signal was used. The values from at least three biological repeats were tabulated and statistically evaluated using GraphPad Prism 6.01.

### Generation of stable transfectants

pLKO.1 TRC cloning vector was used to express the Lyn-shRNA. The sequence with predicted high Lyn knockdown efficiency was obtained from IDT (Coralville, IA) and cloned into the pLKO.1 puro (Addgene # 10878). The sequence was; (F:5′-CCGGGGAATCCTCCTATACGAAATTCTCGAG AATTTCGTATAGGAGGATTCCTTTTTG-3′), (R:5′-AATTCAAAAAGGAATCCTCCTATACGAAATTCTCGAGAATTTCGTATAGGAGGATTCC-3′). The sequence was cloned into pLKO.1 puro following the depositor’s protocol. Lentiviral particles were produced using 2.5 μg pMD2.g (Addgene # 12259), 7.5 μg psPAX2 (Addgene # 12260) and 10 μg with Lyn-shRNA plasmid. HEK293T cells were co-transfected with these plasmids at roughly 50–60% confluence in 10 cm cell culture dishes with DMEM and 10% FBS using TransIT-LT1 transfection reagent (Mirus, Madison, WI) according to the manufacture’s protocol. After 48 h, media containing viral particles was collected and 5 mL of additional media was added to the dish for 24 h until final collection. Total lentiviral containing media was concentrated using Amicon Ultra (Millipore, Billerica, MA) centrifugal filters. Concentrated viral media was stored at –80° C until use. Transduction of HL-60 cells with the lentiviral particles was performed in 6-well plate. 100 μL concentrated viral particles was added to 5 × 10^4^ cells in 1 mL RPMI 1640 with 5% heat-inactivated FBS. After 72 h, transduced cells were transferred into 25 cm^2^ flask and cultured in RPMI 1640 with 5% heat-inactivated FBS and selected for 3 weeks in 0.4 μg /mL puromycin.

### Hierarchical clustering analysis

After densitometrically measuring the nuclear protein expression/activation detected by Western blotting of nuclear lysates from HL-60 myeloblastic cells, we performed hierarchical clustering analysis of expression of the selected proteins from cells that were untreated or treated with ATRA, roscovitine or ATRA plus roscovitine. All normalization, clustering and statistics were performed using R (version 3.6.0; http://www.r-project.org/). Densitometric data on the expression and activation of nuclear molecules for HL-60 cells treated with ATRA, roscovitine and ATRA plus roscovitine were normalized to untreated control. To obtain robust results of clustering and overcome unbalanced distribution of different molecules, z-scores of raw expression values were calculated and used as the clustering algorithm input. The heatmap was generated using the ‘pheatmap’ function in R package ‘pheatmap’ (https://cran.r-project.org/package=pheatmap) [67].

### Statistical analysis

Experiments were biological replicates in triplicate and results were shown as mean and with standard deviation (SD). A two-tailed paired *t* test was used to assess the difference between two groups. A *p* value less than 0.05 was considered to be significant.

## Abbreviations

AML: Acute myeloblastic leukemia
APC: Allophycocyanin
APL: Acute promyelocytic leukemia
ATRA: All-*trans* retinoic acid
BLK: B Lymphoid Tyrosine Kinase
c-Cbl: Casitas B-lineage Lymphoma
CD: Cluster of differentiation
CDK: Cyclin-dependent kinases
ERK: Extracellular Regulated MAP Kinase
Fgr: Gardner-Rasheed feline sarcoma viral (v-fgr) oncogene homolog
GEF: Guanine nucleotide exchange factor
HCK: Hemopoietic Cell Kinase
IRF1: Interferon Regulatory Factor 1
Lck: Lymphocyte-specific protein tyrosine kinase
Lyn v-yes-1: Yamaguchi sarcoma viral related oncogene homolog
MAPK: Mitogen-activated protein kinase
MEK: Mitogen-activated protein kinase kinase
PML: Promyelocytic Leukemia
RAF: Rapidly Accelerated Fibrosarcoma
RAR: Retinoic acid receptor
RARE: Retinoic acid response element
RB: Retinoblastoma
RXR: Retinoid X receptor
shRNA: Small hairpin RNA
SLP-76: SH2 domain containing leukocyte protein of 76kDa
SFK: Src family kinase
